# Assessment of necessity of neuronavigation in localization of calvarial extra-axial lesions in the setting of limited resources

**DOI:** 10.1186/s41016-021-00253-1

**Published:** 2021-08-02

**Authors:** Hussein Soffar, Mohamed F. Alsawy

**Affiliations:** Neurosurgery Department, Kasr Alainy Faculty of Medicine, Cairo University, Al-Saray Street, El Manial, Cairo, Egypt

**Keywords:** Calvarial, Extra-axial, Meningioma, Neuronavigation, Streotaxy

## Abstract

**Background:**

Neuronavigation is a very beneficial tool in modern neurosurgical practice. However, the neuronavigation is not available in most of the hospitals in our country raising the question about its importance in localizing the calvarial extra-axial lesions and to what extent it is safe to operate without it.

**Methods:**

We studied twenty patients with calvarial extra-axial lesions who underwent surgical interventions. All lesions were preoperatively located with both neuronavigation and the usual linear measurements. Both methods were compared regarding the time consumed to localize the tumor and the accuracy of each method to anticipate the actual center of the tumor.

**Results:**

The mean error of distance between the planned center of the tumor and the actual was 6.50 ± 1.762 mm in conventional method, whereas the error was 3.85 ± 1.309 mm in IGS method. Much more time was consumed during the neuronavigation method including booting, registration, and positioning. A statistically significant difference was found between the mean time passed in the conventional method and IGS method (2.05 ± 0.826, 24.90 ± 1.334, respectively), *P*-value < 0.001.

**Conclusion:**

In the setting of limited resources, the linear measurement localization method seems to have an accepted accuracy in the localization of calvarial extra-axial lesions and it saves more time than neuronavigation method.

## Background

Neuronavigation is a very beneficial tool in modern neurosurgical practice. Sometimes, it is nearly impossible to start an operation without its availability especially in small and deep lesions such as glioma and brain metastasis [1].

On the other hand, the necessity of neuronavigation usage is questionable especially in the convexity lesions like meningiomas [2]. Given into consideration the price of the machine and the accompanying sophisticated radiological studies, the enthusiasm becomes weaker to have such technology as a prerequisite to perform a meningioma resection. Unfortunately, the image-guided surgery (IGS) is not available in most hospitals in our country raising the question about its importance in localizing the calvarial extra-axial lesions mainly meningiomas and to what extent it is safe to operate without it.

## Methods

We prospectively studied twenty patients with calvarial convexity meningiomas who would have surgical interventions. The patients were operated upon in the authors’ institute in the period between July 2018 and February 2019.

### Data collection

Preoperative evaluation of all patients was done by history taking, neurological, and radiological assessment. We excluded the recurrent cases from our study. All the patients had a preoperative CT (computed tomography) scan protocol which is compatible with the IGS system. CT scans were performed using a multi-slice CT scanner (Siemens® Somatom Emotion, Erlangen, Germany). MRI (magnetic resonance imaging) study for the brain was done for all the patients before and after administration of IV contrast and MRV (magnetic resonance venography) was done in some cases. MRI was performed using 1.5 Tesla MRI-System (Achieva®, Philips Healthcare, Best, Netherlands). All lesions were preoperatively located with both neuronavigation and linear measurement methods. The intraoperative image guidance was done via StealthStation® S7® System (Medtronic, Inc, Louisville, CO, USA), whereas the usual linear measurements were based on craniometric points.

### Assessment

**Fig. 1:**
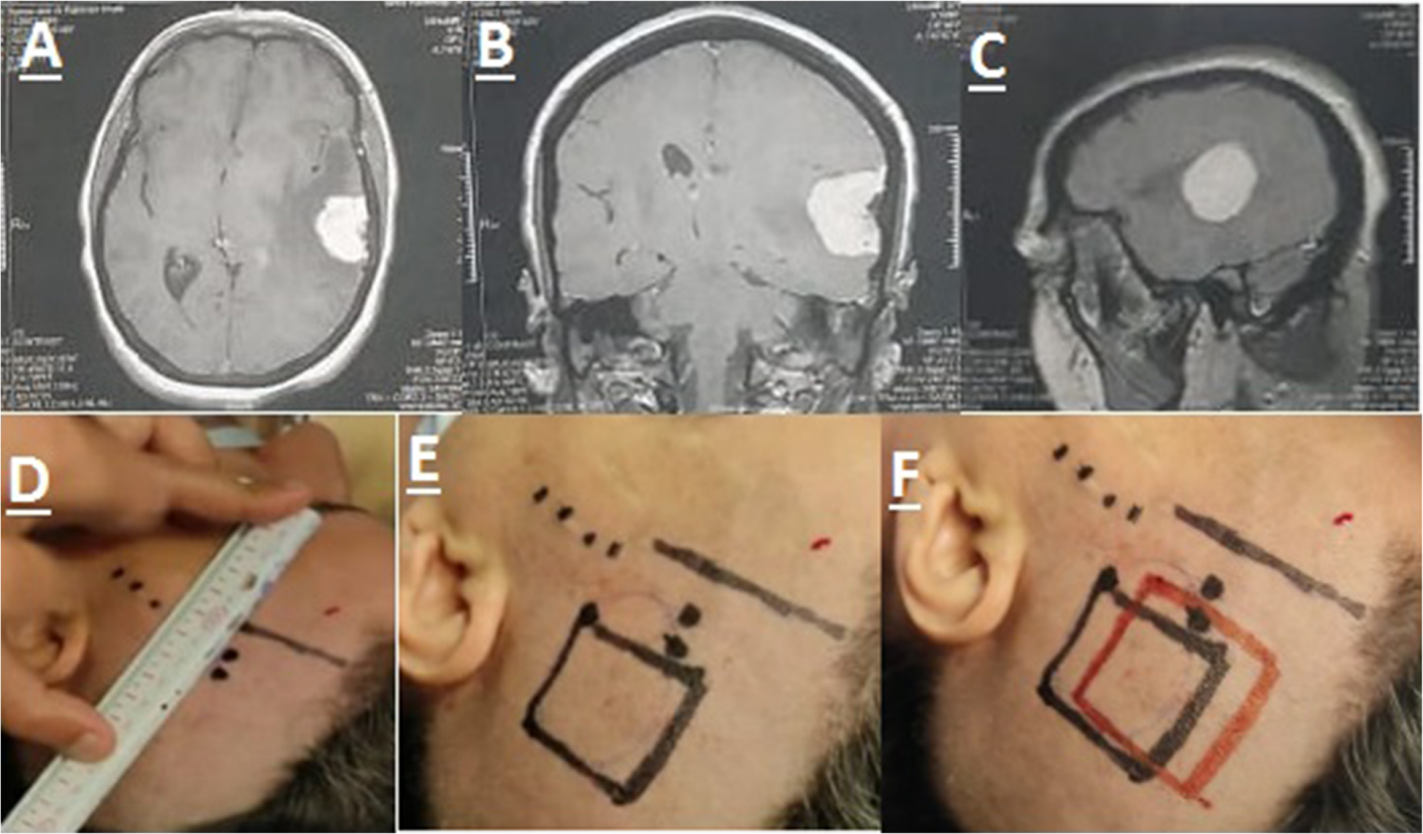
**A-C** Pictures show contrast MRI study for a convexity meningioma case. **D** Picture shows the localization of the tumor margins of the tumor using a ruler to calculate the distance from bony landmarks. **E** Picture shows the localization of the whole tumor using the conventional method marked with the black rectangle. **F** Picture shows the both methods of localization, and the black rectangle is the craniometric localization, while the red rectangle is the navigation assisted localization

Both methods were compared regarding the time consumed to localize the tumor and the accuracy of each method to anticipate the actual center of the tumor. The anatomic localization method is based on the knowledge of the specific bone landmarks of the skull such as coronal suture, external occipital protuberance, and pterion. The localization also depends on the neurosurgeon ability to perform a 3D orientation using the neuroimaging. The expected margins of the tumor were localized using a ruler to calculate the distance from bony landmarks and then those margins were marked on the skin identifying the expected anterior, posterior, medial, and lateral margins of the tumor, then the expected center of the tumor was localized (Fig. 1D, E). The neuronavigator was then used to localize the expected margins and center of the tumor which were marked on the skin with different color (Fig. 1F). After dural opening and full exposure of the tumor, the actual center of the tumor was compared to the expected center in both methods (marked on the skin) and the difference was measured with a tape. The calculated time started immediately after the finishing of anesthetic procedures till planning of an appropriate skin flab was finished. Data was analyzed using paired sample T-test via the Statistical Package of Social Science (SPSS) advanced statistics version 25 (IBM Inc®, Chicago, IL, USA).

## Results

This study included 20 patients with calvarial extra axial lesions. The mean age at the time of surgery for the studied group was 50.6 years ranging from 20 to 70 years. There was a female predominance. There were 14 females (70%) and 6 males (30%) which provide a female/male ratio of (2.33/1). The lesion was left sided in 11 cases (55%) and right sided in 7 cases (35%) and midline in 2 cases (10%). The duration of the presenting symptoms ranged from 2 weeks to 3.5 years with a mean of 8 months. Headache was the most common feature occurring in 75%. Seizures were the main complaint in 20% of cases. Weakness and numbness occurred in 10% of cases and two patients (10%) presented with an altered level of consciousness (Table 1). All the patients had total tumor excision and the pathology was meningioma in all cases.

**Table 1:** Demographics of the patients included in the study with the presenting symptoms and their duration and the site of the lesions

Patient no	Age	Sex	Presentation	Tumor site	Symptoms duration
1	44	F	Headache	Left	14 months
2	56	M	Disturbed conscious level	Right	2 weeks
3	52	F	Headache	Left	9 months
4	61	F	Disturbed conscious level	Left	2 weeks
5	56	M	Headache	Left	6 months
6	20	F	Headache and seizures	Right	3 months
7	48	F	Headache	Midline	24 months
8	47	F	Seizures	Right	2 months
9	42	M	Seizures	Left	5 months
10	39	F	Headache	Right	7 months
11	65	F	Headache	Right	12 months
12	62	M	Headache	Left	10 months
13	41	F	Headache	Left	7 months
14	53	F	Headache	Right	2 months
15	70	M	Headache and weakness	Left	2 months
16	60	F	Headache	Midline	42 months
17	57	F	Headache	Left	5 months
18	66	F	Headache	Left	11 months
19	37	M	Headache and weakness	Left	1 month
20	36	F	Seizures	Right	3 months

A statistically significant difference was found between the mean time passed in the conventional method and IGS method (2.05 ± 0.826, 24.90 ± 1.334, respectively), *P*-value< 0.001. Also, a statistically significant difference was found between conventional method and brain lab one in the prediction of the center of the tumor. The mean error of distance between the planned center of the tumor and the actual was 6.50 ± 1.762 mm in conventional method, whereas the error was 3.85 ± 1.309 mm in IGS method with *P*-value< 0.001 (Table 2).

**Table 2:** Descriptive analysis of the data of the 20 patients regarding accuracy and time consumed in both methods of tumor localization

		Time of conventional (min)	Time of brain lab (min)	Error in conventional (mm)	Error in brain lab (mm)
Mean		2.05	24.90	6.50	3.85
Median		2.00	25.00	6.50	4.00
Std. Deviation		.826	1.334	1.762	1.309
Minimum		1	23	4	2
Maximum		3	27	9	6
Percentiles	25	1.00	24.00	5.00	3.00
	75	3.00	26.00	8.00	5.00
	*P* value	For time < 0.001		For errors < 0.001	

## Discussion

No one can deny the importance of neuronavigation facilities which became a cornerstone in most of the neurosurgical theaters worldwide. In the late eighties of the last century, there was a revolutionary appearance of spatial neuroimaging together with pointing instruments that provided three-dimensional data, which in turn led to the development of “frameless stereotaxy” concept which consequently yielded the expression of “Navigation systems” after improvement of guidance and orientation abilities [3]. Multiple modalities of navigation systems then appeared with increasing efficiency to the extent that they helped the neurosurgeons to plan different procedures interactively, making the approaches less invasive and more accurate especially for subcortical lesions [4–6], but on the other hand, those facilities failed to achieve so much popularity among the society of neurosurgery regarding routine craniotomies [1].

In most cases of calvarial meningiomas and other extra-axial lesions, the neurosurgeon may not be compelled to use neuronavigation since most of the lesions are easily accessible immediately after craniotomy [2]. The only concern that might be worrying in those cases is the size of craniotomy. A suitable size in many opinions is to be slightly larger than the tumor size, giving the surgeons the space needed for dissection of the tumor away from neurovascular structures; on the other hand, a small flab makes the surgery more difficult and more risky, also an extremely larger flab carries the risk of more blood loss and unnecessary exposure of normal neural structures [7].

Despite the expenses of installment of intraoperative navigation system, Paleologos et al. found that cumulative cost for the overall hospital stay is cheaper when the calvarial meningiomas were operated with IGS. They took in consideration a lot of factors including the extra radiological work up that is needed for the navigation guidance and still it was cheaper, they attributed the decreased cost to less ICU (intensive care unit) stay and less postoperative complications in the meningioma patients operated by navigation [2]. But even with this assumption, many tertiary care hospitals in our country lack navigation devices in their operation rooms (OR) due to budgetary difficulties that render purchasing of both hardware and software of the navigation systems primarily, which in turn reduce the dependence on these technologies among the neurosurgeons. Consequently, the neurosurgeons throughout our country, reserve the navigation assistance to operate upon deep and small lesions in more equipped yet few centers. So, we conducted this study to check if it is safe to operate upon calvarial meningiomas without navigation. Although we found a statistical significance between the conventional and IGS methods in anticipating the tumor center, yet we believe that the conventional method is of accepted accuracy and the error difference for each case is trivial as long as the conventional method was carried out by a senior and well trained neurosurgeon. Sun et al. also concluded that surgical planning for parasagittal meningiomas removal can be conducted safely using the craniometric points [8].

Of course, the presence of navigation within OR settings is reassuring to the neurosurgeon; one can feel more confident while operating, knowing that there is an available tool that can help in accurate planning of the surgery with good anticipation for any nearby vascular structures [9, 10], yet the neurosurgeons in the developing countries can bear with more worrying feelings for the sake of curing patients from this benign tumors in the limited resources setting whenever it is safe to operate.

On the contrary, the linear method has a relative advantage over IGS in reducing the overall anesthesia time. We found a statistically significant difference in planning time between both methods; the IGS requires several steps to be functioning probably, whereas the linear method requires much simpler steps in localization with accepted accuracy counting on the experience of the surgical team to translate the radiological studies and to match the tumor location to the patients’ cranium based on the anatomical landmarks. This reduction in anesthesia time could help in decreasing the rate of extracranial complications such as venous thromboembolic episodes [11, 12], postoperative pneumonia [13], and urinary tract infections [14].

## Conclusion

In the setting of limited resources, the intraoperative linear measurement localization of convexity meningiomas and other extra axial lesions seems to be safe and have an accepted accuracy especially when conveyed by an experienced neurosurgeon. Moreover, it saves more time than the neuronavigation method decreasing the incidence of extracranial complications of prolonged anesthesia.

## Data Availability

The datasets used and/or analyzed during the current study are available from the corresponding author on reasonable request.

## Abbreviations

CT: Computed tomography
ICU: Intensive care unit
IGS: Image-guided surgery
MRI: Magnetic resonance imaging
MRV: Magnetic resonance venography
OR: Operation rooms
SPSS: Statistical Package of Social Science
